# Quercetin in the Prevention of Induced Periodontal Disease in Animal Models: A Systematic Review and Meta-Analysis

**DOI:** 10.3390/nu16050735

**Published:** 2024-03-04

**Authors:** Markus Laky, Muazzez Arslan, Xilei Zhu, Xiaohui Rausch-Fan, Andreas Moritz, Anton Sculean, Brenda Laky, Christoph A. Ramseier, Alexandra Stähli, Sigrun Eick

**Affiliations:** 1 Division of Conservative Dentistry and Periodontology, University Clinic of Dentistry, Medical University of Vienna, 1090 Vienna, Austria; 2 Department of Periodontology, School of Dental Medicine, University of Bern, 3010 Bern, Switzerland; 3 Center of Clinical Research, University Clinic of Dentistry, Medical University of Vienna, 1090 Vienna, Austria; 4 Austrian Research Group for Regenerative and Orthopedic Medicine (AURROM), 1050 Vienna, Austria; 5 Austrian Society of Regenerative Medicine, 1010 Vienna, Austria

**Keywords:** quercetin, periodontal disease, meta-analysis, oral health, aging-related diseases, inflammatory response

## Abstract

Background: Periodontitis is an inflammatory condition initiated by oral bacteria and is associated with several systemic diseases. Quercetin is an anti-inflammatory and anti-bacterial poly-phenol present in various foods. The aim of this meta-analysis was the evaluation of the effects of quercetin administration in animal models of experimental periodontitis. Methods: A systematic search was performed in electronic databases using the following search terms: “periodontitis” or “periodontal disease” or “gingivitis” and “quercetin” or “cyanidanol” or “sophoretin” or “pentahydroxyflavone”. In vivo preclinical animal models of experimental periodontal disease with a measurement of alveolar bone loss were included in the analysis. The risk of bias of the included studies was assessed using the SYRCLE tool. Results: The systematic search yielded 335 results. Five studies were included, four of them qualified for a meta-analysis. The meta-analysis showed that quercetin administration decreased alveolar bone loss (τ^2^ = 0.31, 1.88 mm 95%CI: 1.09, 2.67) in experimental periodontal disease animal models. However, the risk of bias assessment indicated that four SYRCLE domains had a high risk of bias. Conclusions: Quercetin diminishes periodontal bone loss and prevents disease progression in animal models of experimental periodontal disease. Quercetin might facilitate periodontal tissue hemostasis by reducing senescent cells, decreasing oxidative stress via SIRT1-induced autophagy, limiting inflammation, and fostering an oral bacterial microenvironment of symbiotic microbiota associated with oral health. Future research will show whether and how the promising preclinical results can be translated into the clinical treatment of periodontal disease.

## 1. Introduction

Periodontal disease is a localized inflammatory condition resulting in the destruction of soft and hard periodontal tissues. Oral hygiene, as well as lifestyle factors like nutrition, smoking, physical activity [1], and systemic conditions like diabetes have an influence on the onset and progression of periodontal disease [2]. Severe periodontitis is the sixth most prevalent disease and affects approximately 743 million people worldwide [3]. The prevalence of periodontal disease increases with age and thus, periodontal disease presents features of age-related inflammatory dysregulation [4]. Animal models have shown that treatment of this altered immuno-inflammatory physiology can also be therapeutically beneficial for periodontal disease [5].

Quercetin is a natural plant-derived dietary polyphenol with a high safety profile and anti-oxidant, anti-inflammatory, and anti-aging effects. The flavonoid quercetin has been part of the human diet for centuries [6]. It is found in various fruits (e.g., apples, cranberries, and grapes) and vegetables (e.g., onion, capers, and traditional herbs) [7,8]. Quercetin acts as a senolytic agent resulting in the activation of mechanisms that eliminate the accumulation of senescent cells, and activates cell apoptosis through a mitochondrial pathway that includes the activation of caspase-3 and caspase-9 by the release of cytochrome C [9,10].

Furthermore, quercetin has a suppressive effect on oral bacteria by damaging the cell envelope, refraining bacterial adhesion, blocking nucleic acid synthesis, and inhibiting pathogen-related biofilms [11]. Zeng et al. [12] demonstrated that quercetin, comparable to chlorhexidine, effectively reduced biofilm dry weight, total protein content, and the number of viable cells in *Streptococcus mutans* biofilms.

The anti-inflammatory effect of quercetin is predominately attributed to its blocking of TNF-α-mediated inflammation in macrophage cells along with its inhibition of cyclooxygenase and lipoxygenase [13,14].

Quercetin is a substance with very distinctive biological activities that can also be used in the treatment of cardiovascular and degenerative neuronal diseases [14]. Quercetin acts as a potent antioxidant by scavenging reactive oxygen species, inhibiting lipid peroxidation, and increasing the activity of antioxidant enzymes. The reduction in oxidative stress in the brain supports protection against neurodegenerative diseases [15]. Additionally, quercetin inhibits the activation of microglial cells and reduces production of proinflammatory cytokines thereby reducing inflammation in the brain and adding to the neuroprotective effects. Quercetin may beneficially affect blood pressure by improving endothelial function, potentially rendering the endothelium less vulnerable to injury and senescence [16]. Quercetin was found to have a hypotensive effect by stimulating autophagy of endothelial cells [17].

Periodontal disease progression is driven by deregulated inflammation and dysbiotic microbiota, hence both clinical conditions might be influenced by the polyphenol quercetin. Mooney et al. [6] reported that quercetin enables sustained periodontal tissue homeostasis in mice and moderates the disease by modulating the inflammatory response and the oral microbial composition. In human macrophage-like cells exposed to lipopolysaccharide and periodontal bacteria, quercetin reduced the cytokine production through its effect on NF-κB signaling [6].

Data showed that quercetin supplementation might facilitate periodontal tissue hemostasis by reducing senescent cells, limiting inflammation, and fostering an oral bacterial microenvironment of symbiotic microbiota associated with oral health. Hence, the objective of this review was to investigate the effects of quercetin administration on the progression of periodontal disease in preclinical periodontitis animal models.

## 2. Material and Methods

### 2.1. Focus Question

The population, intervention, control, and outcome (PICO) framework was used to formulate the focus question.

Population: animals with induced periodontal disease; intervention: quercetin administration; control: no quercetin, vehicle only; and outcome: clinical attachment loss/bone loss.

Type of studies:

Only preclinical studies with quercetin administration in animal models (all species) of periodontal disease were analyzed. The search was not limited by publication date and was restricted to studies published in the English language.

The systematic review followed the “The PRISMA 2020 statement: an updated guideline for reporting systematic reviews” [18].

Search strategy:

An electronic search in four databases was performed up to July 2023: Pubmed, Scopus, Web of Science, Cochrane (Table 1).

**Figure 1:**
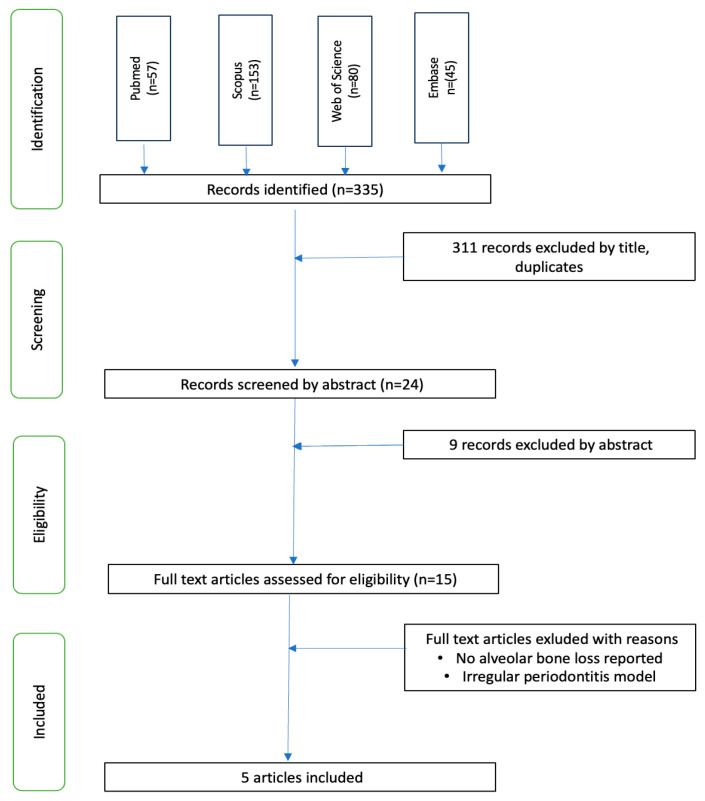
Literature search flow diagram.

The searches in the databases were conducted in accordance with the eligibility criteria (Figure 1). Bone loss data for the experimental groups and the control groups were extracted in order to calculate the change in bone loss due to treatment. Values were taken from tables or estimated from figures in the included articles.

### 2.2. Bias Risk Assessment

The Systematic Review Centre for Laboratory Animal Experimentation (SYRCLE) Risk of Bias tool for animal studies was used to assess the risk of bias. The risk of bias tool for animal studies contains 10 entries. The SYRCLE tool is based on the Cochrane risk of bias tool but has been adjusted for aspects of bias that play a specific role in animal intervention studies [19]. The entries in the SYRCLE tool are related to selection bias, performance bias, detection bias, attrition bias, and reporting bias. The classification of the assessment criteria was conducted by two independent authors (ML, XZ). Any disagreement was resolved in discussion.

### 2.3. Statistical Analysis

Mean alveolar bone loss in the animal models with periodontal disease was examined using RevMan (Version 5.0), Cochrane Center. For this assessment, mean values and standard deviations were utilized. To ensure consistency and minimize methodological heterogeneity, only ligature animal models were included in the meta-analysis. The study by Napimoga et al. [20] using an *Aggregatibacter actinomycetmcomitans*-induced periodontitis model was excluded. Results from the meta-analysis were depicted using a forest plot. A random effect model was applied for the meta-analysis. The statistical heterogeneity among studies was explored by the I^2^ index. Statistical significance was set to *p* < 0.05.

## 3. Results

The search in PubMed, Scopus, Web of Science and Embase returned 335 articles of which 15 were assessed for eligibility in full text. Five were selected for the final analysis. Heterogeneity of the included studies increased considerably with the inclusion of the Napimoga et al. [20] paper. However, the method of the induction of periodontal disease in the article of Napimoga et al. was completely different from the other four articles. The periodontal pathogen *A. actinomycetemcomitans* was used for periodontal disease induction in this paper. The other four articles used a ligature-induced periodontal disease model. Hence, it was concluded that the study by Napimoga et al. [20] was not directly comparable to the other four studies and therefore, only the four publications with ligature-induced periodontal disease were included in the meta-analysis.

Out of the five studies, three used mice, two of them male C57B/J6 mice [6,21]. One study used 6-week-old Sprague Dawley rats [22] and one study used female Wistar rats [14]. The duration of the ligature-induced periodontal disease with and without quercetin ranged from 7 to 15 days. The administration routes of quercetin were oral feeding [22], oral administration [6], oral gavage [21], subcutaneous injection [20], and intraperitoneal injection [14]. Doses varied from 40 to 150 mg/kg per day. All studies evaluated alveolar bone loss (Table 2) and all studies showed reduced bone loss in the quercetin group.

In addition to alveolar bone loss, several other effects of quercetin were reported in the included studies with periodontal disease. Mooney et al. [6] showed a suppression of the NF-κB pathway. In addition to alveolar bone loss, Taskan et al. [14] reported a reduction in MMP-8 levels in the quercetin treatment group. Cheng et al. [22] additionally concluded that quercetin reduced osteoclast formation and periodontal inflammation. Activation of the NRF2 signaling pathway and a reduction in oxidative stress were reported by Wei et al. [21]. In the quercetin group, Napimoga et al. [20] presented a reduction in Il-1β, TNF-α, Il-17, and RANKL compared to control.

### 3.1. Risk of Bias in Studies

The SYCLE tool showed that 60% of the studies had a low risk of bias in the sequence generation. Eighty percent of the included studies demonstrated a low risk of bias concerning “baseline characteristics”, “incomplete outcome data”, and “other biases”. The SYCLE domains “allocation concealment”, “random housing”, “blinding of participants and personnel”, and “blinding of outcome assessment” showed a high risk of bias (Table 3).

### 3.2. Meta-Analysis Results

**Figure 2:**
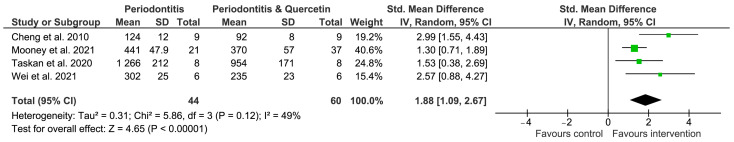
A forest plot of the meta-analysis on alveolar bone loss reduction (mm) for four ligature-induced periodontal disease models [6,14,21,22].

A random effect model was used for the meta-analysis. The statistical heterogeneity among the four included studies showed an I^2^ index of 49%. The meta-analysis showed that quercetin administration significantly decreased alveolar bone loss by 1.88 mm (95%CI: 1.09, 2.67; (τ^2^ = 0.31) in experimental periodontal disease animal models (Figure 2).

## 4. Discussion

In this systematic review and meta-analysis we assessed the effects of quercetin administration on the progression of induced periodontal disease in preclinical animal trials. Of the included studies, all showed less alveolar bone loss compared to the control animals without quercetin administration. The meta-analysis resulted in a significant beneficial effect of quercetin in the prevention of periodontal disease.

Quercetin is a crystalline yellowish substance with a bitter flavor. High levels of quercetin are found in fruits and vegetables like apples, cranberries, grapes, onions, and capers [23]. Depending on food intake and consumption of fruits and vegetables, the dietary intake of quercetin adds up to 50 to 800 mg per day [15]. The poor aqueous solubility of quercetin has limited its pharmacologic availability [24].

The flavonoid quercetin has a high antioxidant capacity and is an effective free radical scavenger [25]. The antioxidant mechanism in vivo of quercetin is due to its effects on glutathione, signal transduction pathways, and reactive oxygen species [26]. It can remove reactive oxygen species and thereby reduce oxidative damage caused by ultraviolet radiation B (UVB) in the skin [27].

Aging is the leading risk factor for age-related diseases like Alzheimer’s disease, diabetes mellitus, osteoporosis, cancer, infertility, and for periodontal disease. The ancient Romans used to say: “Senectus ipsa est morbus” (old age by itself is a disease) [28]. Nutrition is assumed to have an influence over the epigenetic mechanisms, which are effective in the pathogenesis of age-related diseases [29]. Dietary polyphenols have been recognized as efficient agents against aging and aging-related disorders [30]. A diet rich in vegetables and fruits has been found to prevent many diseases and to prolong lifespan. It has also been demonstrated that the beneficial effect on longevity is due to a high content of polyphenols in the diet, indicating a direct relationship between polyphenol consumption and increased lifespan [30]. Recent findings indicate that phytochemicals like quercetin, resveratrol, curcumin, and fisetin regulate the activity of sirtuins [31] [32]. Sirtuins are nicotinamide dinucleotide (NAD+)—dependent deacylases that delay cellular senescence and extend the organismal lifespan [33]. Sirtuins play crucial roles in sustaining genome integrity, by preserving the chromatin condensation state and responding to DNA damage and repair [33]. Identification of SIRT modulators and discovering the functions of these distinctive modulators have provoked increased efforts to ascertain small molecules that can modify SIRT activity [34].

SIRT1 is well-described and the most studied member of the sirtuin family. SIRT1 regulates various cellular functions and is considered as a potential target for aging-associated disorders like Alzheimer´s disease [35], Parkinson´s disease [36], cardiovascular disease, osteoporosis, and diabetes [23,37,38]. Physical exercise results in higher SIRT1 plasma levels [39]. SIRT1 activation by regular physical exercise could possibly be an important factor in reduced periodontal disease progression, as seen in animal models with physical activity [40,41]. Furthermore, SIRT1 plays a vital role in protecting against oxidative stress. Its modulation of transcription factors such as PPAR, NRF, and TFAM increases antioxidant responses and regulates mitochondrial function [31]. By modulating the activity of SIRT1, quercetin affects several pathways like SIRT1/AMPK/NF-κB, SIRT1/PI3K/Akt, and SIRT1/Keap1/Nrf2/Ho-1, which increase the activity of antioxidant enzymes and anti-inflammatory cytokines [23].

Quercetin showed long-lasting and strong anti-inflammatory properties in many tissues [31]. Pro-apoptotic autophagy via the SIRT1/AMPK signaling pathway was found to be induced by quercetin in non-small-cell cancer cell lines in vitro [42].

Natural products like quercetin have the characteristic of acting on multiple targets. SIRT1 and SIRT6 might be of particular importance in relation to periodontitis. Genetic and pharmacologic activation of SIRT6 ameliorates ligature-induced periodontitis by suppressing inflammation and decreasing the number and activity of osteoclasts [43]. However, the data for SIRT6 activation by quercetin are ambiguous. It was shown that quercetin activates SIRT6-induced deacetylation by binding to the SIRT6 selective acyl binding channel at high concentrations [44], but at low concentrations quercetin has been described as an SIRT6 inhibitor [45,46].

An essential aging mechanism that contributes to chronic diseases and age-related dysfunction is cellular senescence [47]. Senescence refers to an irreversible growth arrest of the cells. Cellular senescence is a stress and aging related response that leads to cell cycle arrest in dividing cells. The main proteins in the senescence pathways like p16, p21, and p53 are commonly used as indicators of senescence [48]. These senescent cells are resistant to apoptosis and secrete pro-inflammatory cytokines characterizing the senescence-associated secretory phenotype which leads to local and systemic dysfunction and disease. Quercetin may decrease senescent cells; its administration effectively eliminated senescent human endothelial cells and mouse bone marrow stem cells [49,50].

**Figure 3:**
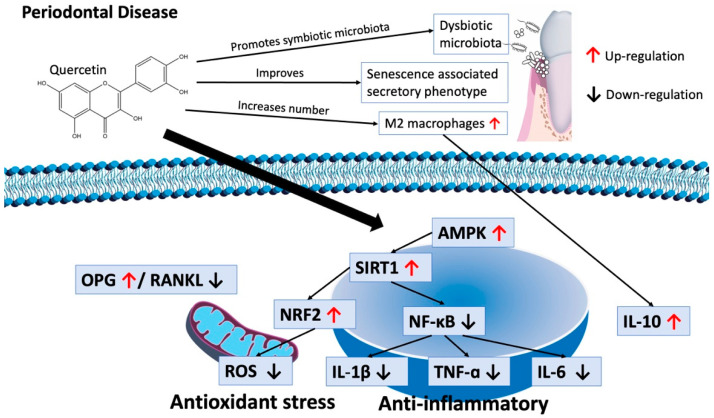
The effects of quercetin in periodontal disease.

Evidence suggests that cellular senescence, stem cell exhaustion, and immunoaging—all hallmarks of natural aging—are related to the diminishing of periodontal hemostasis and the pathophysiology of periodontitis [28]. Reduction in senescent cells might be of interest in the prevention and therapy of periodontal disease (Figure 3). Quercetin might be an option; in an in vivo mouse model it reduced the viability and resulted in cell death of senescent cells [47].

Quercetin ameliorated cigarette smoke related periodontal tissue destruction in mice by reducing oxidative stress damage and autophagy dysfunction [6,51].

Sundaram et al. [52] found that quercetin was able to regulate the expression of various chromatin modifiers and reduce the activity of DNA methyltransferases, histone deacetylases, and histone methyltransferases. Quercetin downregulated overall DNA methylation levels in a dose and time dependent way. Dasatinib in combination with quercetin diminished senescent cell burden and reduced pro-inflammatory cytokine secretion in human adipose tissue [53]. Quercetin, again with additional dasatinib, was shown to clear senescent cells in an atrial fibrillation mouse model and ameliorated the cardiac function. Modulating cell senescence might provide a basis for new therapeutic approaches to atrial fibrillation [48].

Lipophenolic quercetin derivatives reduced the progression of macular degeneration and other age-associated diseases in a mice model [54]. Senescent cells not only play a role in the pathogenesis of periodontitis, they contribute also to the development of peri-implantitis. Yang et al. [55] showed that senescent cells exacerbate peri-implantitis in a peri-implantitis rat model and that the senolytics dasatinib and quercetin reduced implant loss by preventing senescence-related mechanisms. In bone augmentation, ß-tricalcium phosphate granules induced senescence-like cells and the administration of a senolytic combination of dasantinib and quercetin enhanced the bone forming capability of β-tricalcium phosphate [56]. Additionally, a 4% quercetin/polycaprolactone fibrous membrane showed enhanced periodontal bone regeneration in an in vivo rat model compared to an unloaded membrane [57].

Immunosenescence is a progressive modification of the immune system that results in higher susceptibility to infections, cancer, and autoimmune manifestations. Alterations in the function of local cells of the periodontium contribute to the phenomenon of “inflammaging” in periodontal disease [58]. Pathophysiologic changes associated with aging aggravate periodontal disease and are likely responsible for the higher incidence of periodontal disease in older people [5]. Treatment of this senescent phenotype might be therapeutically beneficial and could ameliorate periodontal disease. Bertolini et al. [4] stated that periodontal disease is an age-related chronic inflammatory disease and proposed that periodontal disease might be an effective geroscience model to study mechanisms of age-related inflammatory dysregulation. Age-related changes in immune cells lead to less effective clearance of microbial pathogens and an increase in pro-inflammatory cytokine secretion. Quercetin might contribute to the clearance of senescent cells [59] and the restoration of a more stable periodontal situation.

The antimicrobial mechanisms of quercetin mainly include destroying the cell wall of bacteria and altering the cell permeability, decreasing enzyme activities, and constraining nucleic acid synthesis. Quercetin together with resveratrol reduced the inflammatory process in apical periodontitis, i.e., periapical bone resorption. They increased osteoprotegerin, Il-10 and decreased tartrate resistant acid phosphatase expression compared to control [60]. Ge et al. [61] showed that quercetin increased the number of M2 macrophages, IL-10, and osteoprotegerin and that it reduced RANKL. Quercetin inhibited the growth of microbial pathogens like *Staphylococcus aureus*, *Pseudomonas aeruginosa*, and *Escherichia coli* [25,62]. Quercetin inhibited the formation of *S. aureus* biofilms [63]. Quercetin interfered with pathways involved in bacterial quorum sensing, thus preventing bacterial adhesion and biofilm formation [25,64,65]. Quercetin also inhibited biofilm formation, adhesion, and invasion of *Candida albicans*, and additionally, quercetin ameliorated *C. albicans*-induced inflammation and protected the integrity of the mucosa [66]. In mice, quercetin promoted gut homeostasis by stimulating the populations of *Bifidobacterium* sp. and *Lactobacillus* sp. and inhibiting those of *Enterococcus* sp. and *Fusobacterium* sp. [67].

Quercetin showed antimicrobial effects against *Porphyromonas gingivalis* by damaging the cell membrane. Additionally, quercetin reduced expression of gingipains, decreased hemagglutination, hemolytic activity and virulence gene expression of *P. gingivalis*, and inhibited biofilm formation at subinhibitory concentrations [68]. However, the used concentrations were high and cannot be expected at the site of disease when applied systemically. A local application of quercetin might enhance its concentration and thus the antimicrobial effect in the oral cavity. In a local in vitro setting, a polylactic acid nanofibers membrane loaded with 5–10% of quercetin inhibited subgingival biofilm maturation of *Pseudomonas aeruginosa* and *Streptococcus mutans*. The membrane acted as a reservoir releasing high quercetin concentrations in the presence of oral bacterial infection. Additionally, a reduction in Il-6, Il-1β, and TNF-α release was shown in LPS-stimulated human gingival fibroblasts [69]. Lu et al. [70] showed that quercetin effectively inhibited the secretion of pro-inflammatory cytokines such as Il-1β, TNF-α, and Il-6 in *Mycoplasma gallisepticum* chronic avian respiratory disease and increased the levels of phosphorylated AMPK and SIRT1 while reducing the levels of phosphorylated P65.

Quercetin has a good safety profile. Quercetin, along with its more soluble derivatives, has received approval from the FDA for human use and is generally regarded as safe (GRAS) [71]. However, studies on chronic toxicity in animals, particularly with high doses of quercetin, have indicated an increase in nephrotoxic effects, especially in kidneys that were previously damaged [72]. Dietary polyphenols like quercetin might offer various benefits. They are not a panacea, but when reasonably administered, should be part of a well-adjusted diet. Various foods hold different kinds and amounts of polyphenols and integrating fruits, vegetables, nuts, and grain into the diet will maximize the benefits of dietary polyphenols [73]. It is recommended that the intake of polyphenols like quercetin should be moderate, as disproportionate consumption may not be beneficial and might lead to adverse effects [73].

The focus of the present study was on quercetin tested in preclinical animal experimental periodontitis models. This study has limitations. The SYRCLE risk of bias assessment showed that the domains “allocation concealment”, “random housing”, “blinding of participants and personnel”, and “blinding of outcome” assessment had a high risk of bias. It is important to emphasize that in our thorough literature research, to the best of our knowledge, no comparable research article to our actual research question could be found.

This review suggests a biology model that connects periodontitis and subsequently tooth loss to accelerated biological aging [28].

The results of this meta-analysis demonstrated that bone loss could be attenuated in rats with the administration of quercetin. These findings indicate that quercetin might facilitate periodontal tissue hemostasis by reducing senescent cells, decreasing oxidative stress via SIRT1-induced autophagy, limiting inflammation and fostering an oral symbiotic microbiota associated with oral health.

While data from animal studies are promising, further laboratory and clinical research is needed to clarify the effect of quercetin on the onset, progression, and therapy of periodontal disease. A focus should be placed on formulations and its applications. Future research will show whether and how the promising preclinical results can be translated into the clinical treatment of periodontal disease.

## Figures and Tables

**Table 1:** Search strategy on each database.

Database	Search Strategy
Pubmed	(periodontitis”[MeSH Terms] OR “periodontitis”[Title/Abstract] OR “periodontal diseases” [MeSH Terms] OR “periodontal diseases”[Title/Abstract] OR “gingivitis”[Title/Abstract]) AND (“quercetin” [Title/Abstract] OR “cyanidanol”[Title/Abstract] OR “sophoretin”[Title/Abstract] OR “pentahydroxyflavone”[Title/Abstract])
Scopus	(TITLE-ABS-KEY (periodontitis) OR TITLE-ABS-KEY (gingivitis) OR TITLE-ABS-KEY (periodontal AND disease)) AND (TITLE-ABS-KEY (quercetin) OR TITLE-ABS-KEY (cyanidanol) OR TITLE-ABS-KEY (sophoretin) OR TITLE-ABS-KEY (pentahydroxyflavone))
Web of Science	(TS = (“periodontitis”) OR TS = (“periodontal disease”) OR TS = (“gingivitis”)) AND (TS = (“quercetin”) OR TS = (“cyanidanol”) OR TS = (“sophoretin”) OR TS = (“pentahydroxyflavone”))
Embase	(periodontitis:ti,ab,kw OR ‘periodontal disease’:ti,ab,kw OR gingivitis:ti,ab,kw AND (quercetin:ti,ab,kw OR sophoretin:ti,ab,kw OR cyanidanol: ti,ab,kw OR pentahy- droxyflavone:ti,ab,kw)

**Table 2:** Animal studies on the effect of quercetin on induced periodontal disease.

Author, Year	Animal Model	Periodontitis Model	Time of Ligature	Groups	Quercetin Administration	Dose	Main Outcome
Cheng et al., 2010 [22]	Male 6 weeks old Sprague Dawley rats n = 9 per group	3 O silk ligature	12 days	Control Periodontitis Periodontitis + quercetin	Oral feeding	75 mg/kg	Bone loss: 124 ± 12 µm (P) vs. 92 ± 8 µm (P + Q)
Mooney et al., 2021 [6]	Male, 10–12 week C57B/6J mice n = 37 Quercetin n = 21 vehicle	Silk ligature	7 days	Control, Periodontitis, Periodontitis + quercetin	Oral administration	40 mg/kg/twice per d	Bone loss 441 ± 47.9 µm (P) vs. 370 ± 57 µm (P + Q)
Napimoga et al., 2013 [20]	5 mice per group	Aggregatibacter actinomycetemcomitans JP2 periodontitis model 3 times (0, 48 h, 96 h)	No ligature	Sham Aa infected Aa infected + quercetin	Subcutaneous injection	100 mg/kg 15 days	Bone loss: Aa: 170 µm ± 15 µm Aa + Q: 99 ± 7.6 µm
Taskan et al., 2020 [14]	Female Wistar rats (weighing 230–250 gr.) 8 per group	4–0 silk ligature	15 days	Control Periodontitis Periodontitis + 75 mg/kg/d Periodontitis + 150 mg/kg/d	Intraperitoneal injection 14 days	75 mg/kg 150 mg/kg	Bone loss: P: 1266 ± 212 µm P + 75 mg/kg: 1059 ± 107 µm P + 150 mg/kg: 954 ± 171 µm
Wei et al., 2021 [21]	Male C57BL/6J mice 8 weeks n = 6 per group	Silk ligature	10 days	Control Periodontitis Periodontitis + quercetin	Oral gavage	50 mg/kg	Bone loss bucc.: 302 ± 25 µm (P) vs. 235 ± 23 µm (P + Q) Bone loss pal.: 282 ± 14.5 (P) vs. 216 ± 14.5 mm (P + Q)

**Table 3:** Risk of bias assessment.

Studies	I	II	III	IV	V	VI	VII	VIII	IX	X
Cheng et al., 2010 [22]	+	+	?	?	?	+	?	+	+	+
Mooney et al., 2021 [6]	+	+	?	?	−	+	?	+	+	+
Napimoga et al., 2013 [20]	?	?	?	?	?	?	?	?	+	?
Taskan et al., 2020 [14]	?	+	?	?	+	+	+	+	+	+
Wei et al., 2021 [21]	+	+	?	?	?	+	?	+	+	+

I: sequence generation; II: baseline characteristics; III: allocation concealment; IV: random housing; V: blinding of participants and personnel; VI: random outcome assessment; VII: blinding of outcome assessment; VIII: incomplete outcome data; IX: selective outcome reporting; X: other bias. +: yes (low risk of bias); ?: unclear; −: no (high risk of bias).
